# The use of biologic therapies in uveitis

**Published:** 2018

**Authors:** Ilona Duica, Liliana-Mary Voinea, Costin Mitulescu, Sinziana Istrate, Ioana-Cristina Coman, Radu Ciuluvica

**Affiliations:** * Ophthalmology Department, University Emergency Hospital, Bucharest, Romania; ** “Carol Davila” University of Medicine and Pharmacy, Bucharest, Romania

**Keywords:** uveitis, biologic response modifiers, intravitreal biologic agents

## Abstract

Purpose: Non-infectious uveitis has been long controlled with the use of corticosteroids with many side effects and poor control in some cases. The purpose of this paper was to assess the different biologic agents (in this case infliximab and adalimumab) and to compare their efficacy in the treatment of uveitis.

Results: Adalimumab has been proven very successful in replacing or aiding corticosteroid therapy in different autoimmune mediated uveitis (Juvenile Idiopathic Arthritis, Rheumatoid arthritis, sarcoidosis) whereas infliximab has been used intravenously and recently intravitreally with very promising results in controlling Behcet’s related uveitis.

Conclusion: Biologic Response Modifiers represent the future of therapy in immune-mediated uveitis.

**Abbreviations** AU = Anterior Uveitis, BCVA = Best Corrected Visual Acuity, BRM = Biologic Response Modifiers, CME = Cystoid Macular Oedema, CPR = C Protein Reactive, ESR = Erythrocyte Sediment Rate, HSV = Herpes Simplex Virus, ICAM = Intercellular Adhesion Molecules, IMT = Immunomodulatory Therapy, JIA = Juvenile Idiopathic Arthritis, MMP = Matrix Metalloproteinases, MTX = Methotrexate, RA = Rheumatoid Arthritis, TB = Tuberculosis, VCAM = Vascular Adhesion Molecules

## Background

Ocular inflammatory disorders are associated with visual morbidity and visual loss, resulting in visual worsening (below 20/ 60) in one third of the patients with up to 22% becoming blind [**1**]. They are responsible for up to 10% of blindness in Europe and North America [**2**].

*Corticosteroids* have been traditionally used as first-line therapy [**3**], however, the topical or periocular application of corticosteroids, because of the nature of the disease or local situation, usually does not suffice, and oral corticosteroids are routinely needed. Moreover, the clinical utility of systemic use of corticosteroids is limited by the side effects and is to be avoided in children who have not completed their growth [**4**] and, in many cases, they are not sufficient as monotherapy for chronic uveitis, and do not prevent further relapses, especially in patients with HLA-B27 associated uveitis [**5**].

This has led to a wider use of other *immunomodulatory drugs*, classically sulfasalazine [**6**] or methotrexate [**7**], which have been shown to reduce the need of corticosteroids, decrease ocular inflammation, prevent flares and potential visual loss. Nevertheless, patients can become intolerant or refractory to these classical drugs. This has led in the recent years to the off-label use of biologic agents in patients with non-infectious uveitis that are resistant to classical drugs, showing promising results [**8**].

Based on evidence that shows improvement in long-term visual prognosis and reduction of ocular complications, the Ocular Immunology and Uveitis Foundation recommends the use of immunomodulatory therapy (IMT) as an absolute indication in Adamantiades-Behçet’s disease with retinal involvement, sympathetic ophthalmia, Volg-Konyagi-Harada Syndrome, ANCA associated vasculitides (granulomatosis with polyangiitis, microscopic polyangiitis, polyarteritis nodosa), necrotizing scleritis or peripheral ulcerative keratitis associated with rheumatoid arthritis, relapsing polychondritis, bilateral Mooren’s ulcer or ocular cicatricial pemphigoid. Relative indications for the use of IMT include birdshot retinochoroidopathy, serpiginous choroiditis, sarcoidosis-associated uveitis, severe refractory iridocyclitis, intermediate uveitis, panuveitis, multifocal choroiditis, active retinal vasculitis, and juvenile idiopathic arthritis [**4**].

## Biologic response modifiers (BRM)

The next medication in the “step ladder” approach in the treatment of ocular inflammation, when the antimetabolites fail or are not well tolerated, are biologic response modifiers, also known as “biologics”. The most widely used BRMs for treating uveitis are tumor necrosis factor (TNF) alpha inhibitors, specifically infliximab and adalimumab [**4**].

The cytokine TNF alpha participates in the pathogenesis of autoimmune ocular inflammatory diseases with functions in inflation and apoptosis [**9**]. Animal models have demonstrated that during induction of autoimmune uveitis serum levels of TNF-alpha increase. Increased levels of TNF-alpha were also demonstrated in uveitic patients [**10**]. The inhibition of TNF-alpha was shown to reduce leukocyte rolling, adhesion, and vascular leakage, which might explain the benefic effects in inflammatory ocular disease [**11**].

A recent systematic literature review and meta-analysis of studies published up to March 2016 found evidence that methotrexate, sulfasalazine, cyclosporine A, azathioprine, adalimumab and golimumab might prevent flares, improve ocular inflammation and visual acuity [**12**].

The systematic literature review found evidence for infliximab to be insufficient, because of the lack of sub-analysis and small sample size [**12**]. A recent retrospective cohort study (N=88 patients) found that remission rates of 25%, 50% and 70% were achieved under infliximab at 7, 18 and 45 weeks. However, 58% of the patients needed additional immunomodulatory medication, and 36.4% experienced side effects, with 19% discontinuation secondary to intolerable side effects [**13**]. Although the systematic literature review included two reports on golimumab, the sample size of both studies was small (N=27 patients), and one of those studies reported a non-significant decrease in AU flares [**12**].

In a recent large prospective study of patients with ankylosing spondylitis (N=1250), under adalimumab, the reduction in flares in anterior uveitis (AU) patients was 51% in all study patients, reaching a 68% reduction in patients with a recent history of AU with just 12 discontinuations due to side effects [**14**].

## ADALIMUMAB

Adalimumab is human monoclonal antibody, used initially for patients with rheumatoid arthritis (RA), later on approved for juvenile idiopathic arthritis (JIA), Crohn’s disease, ankylosing spondylitis ulcerative colitis, hidradenitis suppurativa, plaque psoriasis and uveitis starting with the year 2016.

## Pharmacodynamics of Adalimumab

There have been reported cases in wich the use of the drug HUMIRA in patients with rheumatoid arthritis have lowered the acute phase of inflammation reactants such as : C-reactive protein, erythrocyte sedimentation rate and IL-6, compared with baseline patients. The same can be said about patients suffering from Crohn’s disease in wich the C-reactive protein has been found to be lower in patient treated with HUMIRA compared with baseline patients. Another benefit of the treatment is the reduction in serum levels of metalloproteinase such as MMP-1 .

## Pharmacokinetics of Adalimumab

The maximum serum concentration and time to reach that specific concentration in adults are 4.7 ± 1.6 µg/ mL and 131 ± 56 hours respectively. This effect comes from administrating one single subcutaneous 40 mg dose of HUMIRA . The average bioavailability is estimated to be at 64% following one single dose of HUMIRA.[**3**] A linear pharmacokinetic has been noted in doses ranging from 0,5 to 10 mg/kg after one intravenous dose.

The systemic clearance of adalimumab is approximately 12 mL/ hr. The mean terminal half-life was about 2 weeks with a range in numbers between 1- and 20 days. In the synovial fluid the concentration was 31 to 96% of those in serum in patients with rheumatoid arthyritis.

In patients with RA that were given 40 mg HUMIRA every other week without and with methotrexate (MTX), the adalimumab mean steady-state trough concentrations of 5-9 µg/ mL. In patients with rheumatoid arthritis MTX reduced HUMIRA’s clearance after one&more dosing by 29%-44%. 

In comparison to the concentrations in rhemautoid arthritis patients with psoriatic arthritis treated with HUMIRA had a mean steady-state trough concentrations slightly higher (6 -10 µg/ mL and 8.5 -12 µg/ mL, without and with MTX, respectively) . The same can not be said about patients with ankylosis spondylitis where the pharmacokinetic properties do not differ with those of RA patients [**16**].

The presence of anti-adalimumab antibodies determines a higher elimination of the substance and a lowering of elimination has been found to be related with aging ranging from 40-75 years.Other factors such as high C protein levels or sub-dose administration have been reported to increase clearance

No gender-related pharmacokinetic differences were noted.No differences have been found in pharmacodynamic between healthy and RA patiens . Regarding hepatic or renal problems there are no studies.

## Adalimumab in uveitis

Visual I, a large (adalimumab arm=110, placebo arm=107) multinational randomized, placebo-controlled masked phase 3 trial presented a noteworthy lower risk of treatment failure [**17**].

The same effect was proven in Visual II (adalimumab arm=115, placebo arm=115), which included patients with *inactive* non-infectious intermediate, posterior or panuveitis [**18**].

Visual I and Visual II had identical therapy strategies with a intial dose of 80mg then carried with a dose of 40mg every two weeks. Following the results from these two studies the FDA approved the use of adalimumab in treating non-infectious intermediate, posterior and panuveitis [**15**].

Patients who participated in Visual I and Visual II were eligible to participate in a multicenter extension study (VISUAL III), where preliminary data indicate that uveitis remains well controlled by adalimumab over the longer term [**19**].

## Adalimumab in specific subsets of uveitis patients

**• Ankylosing spondylitis**

In a recent large prospective study of patients with ankylosing spondylitis (N=1250) under adalimumab, the reduction in flares in anterior uveitis (AU) patients was 51% in all the study patients, reaching a 68% reduction in patients with a recent history of AU with just 12 discontinuations due to side effects [**14**]. A smaller (N=77 patients) study from the Netherlands, that followed the patients for 1.7 years, showed an 84% reduction in flares by recently diagnosed uveitis, and 80% reduction in flares in all the patients [**20**].

**• Sarcoidosis**

A prospective study that enrolled 26 patients with refractory uveitis to prednisone and/ or methotrexate showed a total resolution of papillitis, an improvement in all cases of CME, a partial or total improvement of choroiditis. This was also the case with vitritis and vasculitis. The patients also showed a significant reduction in systemic corticosteroids and methotrexate use [**21**].

**• Behçet disease**

Adalimumab was studied in a cohort of 11 male patients (21 eyes) over 10.8 months, with promising results: improvement in visual acuity in all but 4 eyes, two of which had macular scars and a 43% reduction in corticosteroids [**22**].

**• Juvenile idiopathic arthritis**

Although it is not FDA approved for use in children with uveitis, up to 38% of the children with juvenile arthritis are affected by uveitis, leaving those of approximately 15% legally blind [**23**]. This has led to a multicentre double-blinded, randomized, placebo-controlled study that enrolled 90 patients who had a stable dose of methotrexate and were assigned either to a placebo group or to receive adalimumab at a dose of 20mg or 40mg, according to body weight. The adalimumab arm had a significantly less treatment failure than the placebo arm (27% vs. 60%). However, adverse events were reported more frequently in the adalimumab arm [**23**]. This effect was also proven in 5 other retrospective studies [**15**].

**Safety**

The most common side effects are localized site reaction (pain, erythema, and rash).

The industry supported phase 3 open label continuation studies with a mean duration of exposure of 6.2 years, showing the most frequent adverse events to be upper respiratory infection (46%), disease flare (34%), and sinusitis (25%). Malignancies were reported in 14.8% of the patients including leukemia, lymphoma, and melanoma. Most of these were deemed unlikely related to the study drug [**24**].

A study including 131 patients reported adverse events such as fatigue, hypertension, zoster, infectious mononucleosis, hepatitis C reactivation, and uveitis reactivation [**25**]. Other rare described side effects include cerebrovascular accident, hypoglycemic coma, conversion to ANA positivity, headache, joint pain, nausea, common cold, retinal detachment, skin rash, carcinoid tumor of gastrointestinal tract, glioblastoma multiforme, active TB (tuberculosis), latent TB, lupus-like reaction, demyelinating disorders, anti-adalimumab antibodies, pulmonary sarcoidosis, bronchitis, neutropenia, worsen migraine, hepatic stenosis, HSV (herpes simplex virus) keratitis, pneumonia, anaemia, depression, mildly elevated serum transaminases, secondary adrenal insufficiency, otitis media, candida vaginitis, gastritis and psychiatric disease [**15**].

## Conclusion

All the evidence pointed to Adalimumab as the best immunomodulatory drug, which ultimately led to its approval by the US Food and Drug Administration (FDA) as the only non-corticosteroid agent for the treatment of non-infectious uveitis. Although it is only approved in the US for use in intermediate, posterior and panuveitis in adults, a recent expert review recommended its use also in off-label treatment of paediatric uveitis and scleritis [**15**].

## INFLIXIMAB

Infliximab is a chimeric monoclonal IgG1 antibody and tumor necrosis factor (TNF-alpha or TNF-α) blocker. Tumor necrosis factor-alpha (TNF-α) is an important proinflammatory cytokine associated with chronic inflammatory diseases. Its high activity and augmented signal producing pathways are found in inflammatory diseases where it determines the further activation of other pro-inflammatory components. Because it binds to both the soluble subunit and the membrane-bound precursor of TNF-α infliximab causes a disruption of the interaction of TNF-α with its receptors and can determine TNF-alfa producing cell apoptosis .

Infliximab was first approved in 1998 under the production name Remicade, as an intravenous injection. It is useful in treating many inflammatory diseases such as adult or paediatric Chron’s disease, adult, or paediatric ulcerative colitis, rheumatoid arthritis in combination with methotrexate, ankylosing spondylitis, psoriatic arthritis, and plaque psoriasis.

## Pharmacology of Infliximab

It is useful in: 

• lessening symptoms and signs and creating&maintaining clinical remission in adult or paediatric (≥ 6 years of age) patients with moderately to severely active Crohn’s disease, that have had a poor response with conventional therapy.

• in adult patients with fistulizing Crohn’s disease it has lowered the rate of draining enterocutaneous and rectovaginal fistulas and maintained fistula closure 

• lessening symptoms and signs, creating& maintaining clinical remission, and removing corticosteroid use in adult or paediatric (≥ 6 years of age) patients with moderately to severely active ulcerative colitis, that had a poor response to the usual therapy.

• in patients with moderately to severely active rheumatoid arthritis in combination with methotrexate, infliximab has reduced the signs and symptoms, hindered the progression of structural damage and improved physical function 

• in patients with active ankylosing spondylitis it has lessened the signs and symptoms 

• in patients with psoriatic arthritis it has reduced signs and symptoms of active arthritis, hindered the progression of structural damage, and improved physical function.

• the treatment of adult patients with chronic severe plaque psoriasis, that have indication for systemic therapy and when other systemic therapies are medically less suitable.

## Pharmacodynamics of Infliximab

Infliximab interrupts the signals in the inflammatory cascade cycle. Infliximab lowers the infiltration rate of cells at the inflammation site. It also attenuates the expression of molecules mediating cellular adhesion {including E-selectin, intercellular adhesion molecule-1 (ICAM-1)}, and vascular cell adhesion molecule-1 (VCAM-1)}, chemoattraction {[IL-8 and monocyte chemotactic protein (MCP-1)} and tissue degradation {matrix metalloproteinase (MMP) 1 and 3}.

## Safety

Common side effects of infliximab include upper respiratory tract infections, urinary tract infections, cough, rash, back pain, nausea, vomiting, abdominal pain, headache, weakness and fever. Other noted side effects include low or high blood pressure, chest pain, difficulty breathing, rash, itching, fever and chills [**27**].

Such side effects could indicate an allergy to infliximab. Patients that develop infliximab antibodies have a greater risk of side effects and patients taking drugs that suppress the immune system have a lower rate of side effects.

Infliximab should be stopped if serious reactions occur. Dangerous infections have been documented with other drugs that block TNF-alpha, and infliximab is no different. Infliximab should be stopped if a severe infection develops during treatment. Testing for tuberculosis (PPD tests for TB) should be done before treatment because of reports of reaccurence of tuberculosis in patients that were treated with infliximab. Low values of leucockytes, erythrocites and platelet counts have been reported with infliximab. Some cases of vasculitis have been reported also.

## Infliximab and uveitis

**Behcet’s uveitis**

Infliximab has been proven to be effective in Behcet associated uveitis [**26**]. Suhler et al. [**28**] have proven this in his study concerning Behcet associated uveitis, a study that took place using 23 patients with Behcet disease with refractory uveitis. Patients received 3 infliximab infusions at 0, 2 and 6 weeks at a dose of 3 mg/ kg if given in addition to other immunosuppressive medications, or at 5 mg/ kg if infliximab was given as monotherapy. Patients who had positive results to treatment on week 10 were given a further infusion on week 14 and then every 8 weeks up to the finalization of the study at 50 weeks. The efficacy of the infliximab therapy was separated according to 4 conditions: final visual acuity, control of intraocular inflammation, improvement in inflammatory signs on fluorescein angiography or optical coherence tomography, and capacity to reduce other anti-inflammatory medication. Treatment was considered effective if one of the parameters improved or if none of them exacerbated; effectiveness was reported in 18 out of 23 patients at the 10-week follow up point.

In the study, 4 patients manifested improvement in 2 of the 4 specifications and one had 3 specifications improved (only 1 patient in the study had visual improvement). Because of the side effects ( liver or heart failure) some patients could not complete the study.

Markomichelakis et al. [**29**] have analysed the efficacy in reducing ocular inflammation between infliximab and triamcinolone and have proven that infliximab is superior in effect. In the infliximab group, vasculitis has been reduced from 79% to 15% at the 14 days follow-up compared to 100% and 87.5% respectively in the intravitreal triamcinolone acetonide group. Hamza et al. coordinated a study [**30**] in which one single injection of infliximab was prescribed to patients with Behcet disease refractory uveitis. By the 18 weeks follow-up, they noted a significant improvement in visual acuity, downsizing in mean central macular thickness, and scaling down of mean vitreous haze score. In 2008, the European League Against Rheumatism (EULAR) Committee issued the recommendations for the management of BD, in which they support the use of infliximab for patients with severe eye disease [**31**,**32**]. Patients with BD associated eye disease should be started on a treatment method that includes systemic steroids and azathioprine, and infliximab or cyclosporine should be added in serious cases. Anti-histamine medication has lowered the number of adverse reactions in infliximab therapy. One of the most dreadful side effects is the reactivation or primary TB infection, therefore patients should be screened for undiagnosed TB before beginning biologic therapy. Biologic agents should be used with caution in patients prone to recurrent opportunistic infections and should be monitored closely [**34**-**38**].

## Intravitreal Infliximab

In animal models suchs as rabbits and primates of single dose of intravitreal infliximab injection has been proven safe. These studies [**29**,**33**] have shown no indications of intraocular inflammation or toxicity by clinical, electrophysiological, and histopathological examination for up to 90 days even with three repeated monthly injections. But, the study conducted by Rassi et al. [**39**] was the only one to account the severe intraocular inflammation in 1 eye out of 12 rabbit eyes at 90 days following three intravitreal injections (2 mg monthly). The half-life of intravitreal infliximab was established to be 6.5 days to 8.5 days, and it is also able to penetrate all retinal layers. After the publication of these studies one could have concluded that it is safe to use infliximab in intravitreal injection form. Sadly, some serious side effects have been found in clinical studies conducted this far showing various and inconsistent results in terms of the safety and efficacy of intravitreal infliximab. These studies were organized on patients with refractory as well as naive cases of age related macular degeneration, choroidal neovascularization, diabetic, central retinal vein occlusion, macular edema , pseudophakic macular edema, angiomatous malformations and uveitis. The doses used extended from 0.5 mg to 2 mg. Two studies [**40**] have tracked injected eyes with flash electroretinogram (ERG) with mild reversible effects at 3 months in one study (2 age-related macular degeneration eyes and two diabetic macular edema eyes using 0.5 mg), and no effects in the second study(six age-related macular degeneration eyes using 2 mg) at 6 months [**41**,**42**].

The initial study by Theodossiadis et al. in 2009 [**34**] did not account any intraocular inflammation in three patients undergoing two intravitreal injections of 1 mg and 2 mg for refractory age-related macular degeneration choroidal neovascularization with 7 months follow-up interval. Later several clinical studies [**37**,**38**,**40**,**41**] have reported serious intraocular inflammation following intravitreal injections of infliximab in non-uveitic patients. This happened with doses as low as 0.5 mg, in 15% to 100% of the patients, and usually starts developing several weeks after the intravitreal injections. Caution should be taken when administrating intravitreal infliximab given these recent facts. However, studies investigating intravitreal infliximab in uveitis patients have shown enhancement in vision, lessening in central foveal thickness (CFT), on optical coherence tomography (OCT), and reduction in inflammation with no reported adverse effects [**40**]. The well-being and success of three intravitreal infliximab injections in a row (1 mg/ 0.05 mL, 6 weeks apart) in a selected group of patients with refractory uveitis in Behcet’s disease have been explored in a study [**30**].

All parameters of intraocular inflammation showed significant improvement during the study period[**30**]. The maximum drug effect was reached at 4 weeks. Vitreous haze reached its lowest rate at 4 weeks, and only two patients had vasculitis, and no patient had retinitis or papillitis at 4 weeks. There was mild increase of vitreous haze between 4 and 6 weeks, and between 8 and 12 weeks, with rapid control of activity after the reinjections of the second and third injections, respectively. The result of the study confirms the favorable visual and anatomical outcomes of intravitreal infliximab that were reported by Farvardin et al. and Markomichelakis et al. on eyes with uveitis [**29**]. All previous three studies [**30**,**43**,**44**] have shown significant control of intraocular activities within weeks with significant improvement of BCVA and reduction of macular edema by OCT. Based on the results of the study [**30**] and previous clinical studies [**43**,**44**], intravitreal infliximab appeared to be very effective in cases of posterior non-infectious uveitis including Behcet’s disease. The intravitreal infliximab dose of 1 mg was well tolerated with no observed adverse effects clinically and by flash ERG. However, these effects were temporary, and reinjections were needed perhaps at intervals shorter than 6 weeks.

## Conclusions

There is an innovative approach in treating non-infectious uveitis with this new form of therapy using biologic agents and furthermore using them intravitreally. TNF can also have a part in the pathogenesis of macular edema and some studies report good outcome with using intravitreal anti-TNF in the treatment of diabetic macular edema and neovascular age related macular degeneration. Although the intravitreal route has fewer systemic side effects, for wide clinical application, more studies are warranted.

**Conflict of interest**

The authors declare that there is no conflict of interest. The authors have equally contributed to this paper.
